# Stochastic expression of a multiple antibiotic resistance activator confers transient resistance in single cells

**DOI:** 10.1038/srep19538

**Published:** 2016-01-13

**Authors:** Imane El Meouche, Yik Siu, Mary J. Dunlop

**Affiliations:** 1 School of Engineering, University of Vermont, Burlington, VT USA 05405

## Abstract

Transient resistance can allow microorganisms to temporarily survive lethal concentrations of antibiotics. This can be accomplished through stochastic mechanisms, where individual cells within a population display diverse phenotypes to hedge against the appearance of an antibiotic. To date, research on transient stochastic resistance has focused primarily on mechanisms where a subpopulation of cells enters a dormant, drug-tolerant state. However, a fundamental question is whether stochastic gene expression can also generate variable resistance levels among growing cells in a population. We hypothesized that stochastic expression of antibiotic-inducible resistance mechanisms might play such a role. To investigate this, we focused on a prototypical example of such a system: the multiple antibiotic resistance activator MarA. Previous studies have shown that induction of MarA can lead to a multidrug resistant phenotype at the population level. We asked whether MarA expression also has a stochastic component, even when uninduced. Time lapse microscopy showed that isogenic cells express heterogeneous, dynamic levels of MarA, which were correlated with transient antibiotic survival. This finding has important clinical implications, as stochastic expression of resistance genes may be widespread, allowing populations to hedge against the sudden appearance of an antibiotic.

Bacteria can evade antibiotics through transient expression of resistance genes. By temporarily elevating resistance in a subset of cells, a population can undermine the efficacy of antibiotics resulting in chronic and recalcitrant infections^1^,^2^. For example, in bacterial persistence a small fraction of cells (≤1 in 100) called persisters stochastically enter a dormant, drug-tolerant state, allowing the population to hedge against the sudden appearance of an antibiotic^3^,^4^. It is important to recognize that transient resistance is not caused by genetic changes, rather cells use phenotypic variability or induce gene expression to generate a resistant phenotype^2^,^3^,^5^,^6^. To date, research on phenotypic variability in antibiotic resistance has focused primarily on dormancy. However, little is known about transient resistance strategies that generate a continuum of resistance levels within growing cells.

Isogenic bacterial populations are traditionally considered to be composed of identical cells. However, even though individual cells contain the same genetic material, protein levels between cells can vary due to stochastic events associated with gene expression and regulation^7^. Cell-to-cell heterogeneity has important implications, allowing populations of cells to diversify in order to survive environmental stress^8^, and evade the immune response^9^,^10^.

In contrast to permanent antibiotic resistance, usually associated with mutations or acquisition of resistance elements via horizontal or vertical transfer^11^, transient resistance allows cells to temporarily survive the appearance of an antibiotic. For instance, the pathogen *Mycobacterium tuberculosis* has subpopulations of non-replicating cells characterized by high antibiotic tolerance^12^,^13^,^14^. Bacterial persistence is a well-studied example of stochastic variation that results in a small fraction of cells that can survive antibiotic stress^3^,^15^. In persistence, cellular mechanisms such as DNA and protein synthesis are inhibited and consequently cells remain dormant and evade antibiotics that target cell growth processes^15^. An increasing number of studies have determined key factors involved in persistence. Toxin-antitoxin systems are often involved and quantitative measurements at the single cell level have shown that overexpression of the toxin can determine when and for how long a cell will remain in the dormant state^6^. In addition, stringent response via the mediator (p)ppGpp, reduced membrane potential, and extended single-cell lag times can confer transient antibiotic resistance^16^,^17^,^18^. These mechanisms have the common feature that antibiotic tolerance is achieved by temporarily placing cells in a dormant state.

Other studies suggest that mechanisms for transient antibiotic resistance exist beyond dormancy, however the molecular basis often remains unclear. A study in *Escherichia coli* showed that stress-induced variability within an isogenic population is transmissible between generations and plays a role in antibiotic survival at the single cell level, possibly by modifying membrane permeability^19^. Furthermore, cell populations can differentiate into resistant subpopulations with variable growth statuses due to cephalosporin hydrolase expression^20^. In *Salmonella enterica*, heterogeneous levels of porins and efflux pumps contribute to differential levels of antibiotic resistance^21^. In addition, single cell studies have shown that the probability of *E. coli* cell lysis correlates with the time since the last cell division^22^. Asymmetric cell division events^23^ and stochastic pulses in the catalase-peroxidase KatG^24^ in mycobacteria result in differences in antibiotic susceptibility. These studies hint at additional pathways by which cells can use stochastic, non-genetic variability to survive antibiotics.

In addition to stochastic effects, cells can evade antibiotics by transiently inducing antibiotic resistance at the population level^25^,^26^. A well-studied example of this is expression of MarA (the multiple antibiotic resistance activator), which plays a key role in multidrug resistance in enteric bacteria^27^. In *E. coli*, MarA expression can be induced by the addition of extracellular compounds, including antibiotics^28^,^29^,^30^. Thus, when antibiotics are detected, resistance genes are turned on, leading to population-wide resistance. MarA expression is regulated by a combination of positive and negative feedback loops (Fig. 1a). The *marRAB* operon is autoactivated by MarA and autorepressed by MarR^31^,^32^; the periplasmic protein MarB also indirectly represses *marRAB* expression^33^. MarA activates over 40 downstream genes implicated in antibiotic resistance^34^. Examples include *micF*, an antisense RNA that represses the expression of the outer membrane porin OmpF, and the *acrAB*-*tolC* multidrug efflux pump genes^34^.

In this work, we focused on the role of MarA in transient resistance. Although it is well-known that MarA can induce antibiotic resistance at the population level, we asked whether stochastic expression of MarA could provide antibiotic resistance at the single cell level, even when uninduced. Using time-lapse microscopy, we studied MarA dynamics in isogenic cells and found cell-to-cell variability in MarA expression, which correlated with antibiotic susceptibility. This phenotypic variation has the potential to generate diverse resistance phenotypes within a population.

## Results

### MarA overexpression increases antibiotic resistance in population measurements

**Figure 1:**
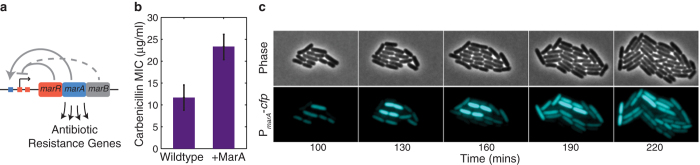
Cell-to-cell variability in the multiple antibiotic resistance activator MarA. (**a**) Schematic view of the *marRAB* operon. MarA activates the operon by binding to one site within the operator, MarR represses its expression by binding to two sites, and MarB indirectly represses expression of the operon. (**b**) Minimum inhibitory concentration^48^ of carbenicillin for the strains P_*marA*_*-cfp* (wildtype) and MarA-CFP (+MarA). Error bars show standard deviations from three biological replicates. (**c**) A representative filmstrip of time-lapse microscopy images showing variability in P_*marA*_*-cfp* fluorescence levels within a microcolony. Supplementary Movie 1 shows additional details.

MarA’s role in activating downstream multidrug resistance genes has been studied extensively at the population level^27^,^28^,^29^,^30^,^31^,^32^. In this work we used carbenicillin, a bactericidal antibiotic that inhibits cell-wall synthesis^35^. We first measured the minimum inhibitory concentration of carbenicillin in *E. coli* MG1655 and in the same strain with a plasmid overexpressing MarA (Fig. 1b). Consistent with previous reports, overexpression of MarA increased antibiotic resistance^36^.

### MarA expression is heterogeneous at the single cell level

Although inducible population-level resistance is well established, we wondered whether MarA expression is variable at the single cell level. Previous computational studies by our group have hypothesized that the feedback structure regulating MarA can produce stochastic MarA expression when the system is uninduced^37^. Motivated by these computational predictions, we experimentally measured the dynamics using a plasmid that reports MarA levels in the cell. To do this, we used a modified version of the *marRAB* promoter containing transversion mutations that inactivate the MarR binding sites in the operator, leaving the MarA binding site intact^29^. We fused this promoter to a cyan fluorescent protein gene (*cfp*) with an *ssrA* degradation tag to decrease the protein half-life and increase temporal resolution^38^. We conducted experiments with this plasmid in *E. coli* MG1655 (we refer to this strain as P_*marA*_-*cfp*). We note that MarR binding sites were only removed in the reporter plasmid; the chromosomal copy of the *marRAB* promoter remained unchanged. The promoter modification in the reporter was necessary to visualize CFP and allowed us to measure MarA independent of the action of MarR. In order to study dynamics and heterogeneity in MarA expression at the single cell level, we conducted time-lapse microscopy experiments with P_*marA*_-*cfp*. Within growing microcolonies we observed heterogeneous MarA expression that fluctuated over time (Fig. 1c and Supplementary Movie 1). Therefore, MarA expression is stochastic within single cell lineages.

### MarA variability is correlated with survival in the presence of carbenicillin within an isogenic *E. coli* population

**Figure 2:**
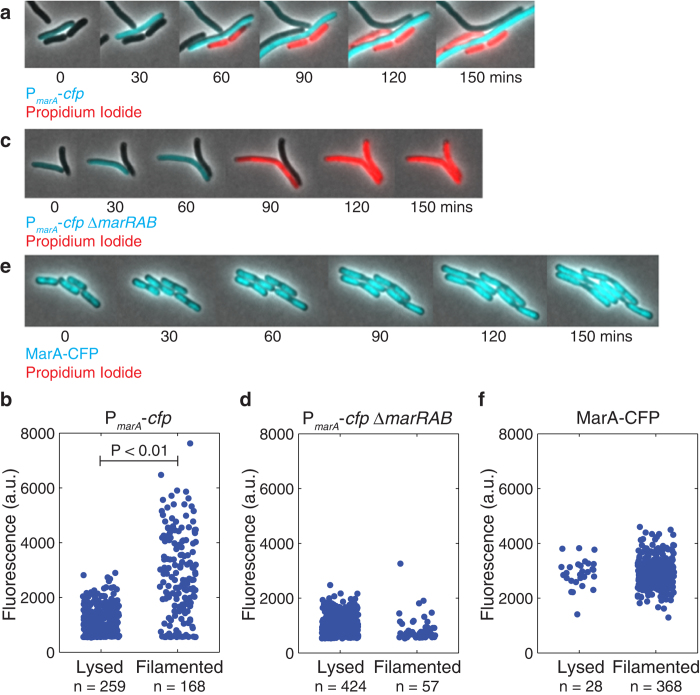
Variability in MarA expression is correlated with a heterogeneous response to carbenicillin treatment. (**a**,**c**,**e**) Time-lapse microscopy images of (**a**) P_*marA*_*-cfp*, (**c**) P_*marA*_*-cfp* Δ*marRAB*, and (**e**) MarA-CFP in the presence of 50 μg/ml carbenicillin and 10 μg/ml propidium iodide. Cells were introduced onto agarose pads containing carbenicillin and propidium iodide at t = 0 mins and imaged over the course of 400 mins in two color channels. Cyan indicates CFP levels from the MarA reporter; red indicates the death marker propidium iodide. Supplementary Movie 2 shows additional details for the P_*marA*_*-cfp* strain. Note that in the MarA-CFP strain the localization patterns in CFP are due to binding of MarA to DNA. (**b**,**d**,**f**) Outcomes of individual cells after 400 mins of carbenicillin exposure, plotted versus CFP fluorescence at t = 0 mins for (**b**) P_*marA*_*-cfp*, (**d**) P_*marA*_*-cfp* Δ*marRAB*, and (**f**) MarA-CFP. Each blue dot corresponds to one cell, which has an outcome ‘lysed’ or ‘filamented’ and an initial fluorescence value. The number of cells exhibiting each outcome is listed on the x-axis. The mean ranks are statistically different for only the P_*marA*_*-cfp* strain (P < 0.01 by a Mann-Whitney rank sum test). Histograms and further details are provided in Supplementary Fig. 4.

We next asked whether variability in MarA expression impacts survival under antibiotic treatment at the single cell level. Bacteria can transiently defend against antibiotic lethality by inducing the SOS response, which inhibits bacterial cell division but not elongation, enabling survival in the presence of lethal concentrations of antibiotics^39^. We exposed cells containing the MarA reporter P_*marA*_-*cfp* to lethal concentration of carbenicillin (50 μg/ml) on agarose pads and observed the impact on individual cells using time-lapse microscopy (Fig. 2a and Supplementary Movie 2). Cells lysis occurred rapidly after incubation with carbenicillin for a subset of cells in the population. As an indicator of cell death we used propidium iodide, which enters the cells and stains DNA if the membrane is depolarized^40^. Using P_*marA*_-*cfp*, we measured the initial fluorescence level of each cell at t = 0 mins. We then recorded the outcome of each cell after 400 mins of carbenicillin exposure (Fig. 2b). This duration, sufficient to kill a significant fraction of *E. coli* cells, allowed us to ensure that transient effects due to cell division time were not a factor in our analysis^22^. We primarily observed two outcomes: cell lysis, as indicated by propidium iodide staining, and filamentation, where cells elongate but do not lyse. A small fraction (~10%) of cells neither stained with propidium iodide nor formed filaments and were excluded from subsequent analysis. Each cell outcome was assigned to the initial CFP value reflecting the MarA expression level in the cell at t = 0 mins. As expected from our time-lapse microscopy experiments (Fig. 1c), we observed a distribution of initial fluorescence levels corresponding to cell-to-cell variability in MarA expression (Fig. 2b). We also observed a heterogeneous response to carbenicillin. Interestingly, heterogeneous outcomes were correlated with MarA variability between isogenic cells, where cells that filamented were more likely to have high initial MarA levels (Fig. 2b).

To determine if variability in fluorescence levels was due to MarA expression itself, we introduced the same fluorescent reporter into a strain lacking the *marRAB* operon (we refer to this as P_*marA*_-*cfp* Δ*marRAB*). We recorded initial fluorescence levels and cell outcomes in the presence of carbenicillin as before (Fig. 2c,d). Cells exhibited higher lysis rates following carbenicillin exposure than in the strain with the intact *marRAB* operon. CFP levels for P_*marA*_-*cfp* Δ*marRAB* were lower than for P_*marA*_-*cfp*, as expected given the absence of MarA.

As a positive control, we also constructed a MarA-CFP protein fusion in order to produce a population with high, homogeneous expression of MarA. The translational fusion stabilizes MarA, increasing its half-life to ~30 mins (Supplementary Fig. 1), in contrast to ~1 min for wildtype MarA^41^. As a result, cells exhibited homogeneous fluorescence levels (Fig. 2e). It is important to note that the CFP levels for this strain do not report the same levels of MarA as those strains with P_*marA*_-*cfp*. Instead, the MarA-CFP strain has markedly higher levels of MarA than either the P_*marA*_-*cfp* or P_*marA*_-*cfp* Δ*marRAB* strain due to the stabilized protein. When we exposed cells with MarA-CFP to carbenicillin, we observed a dramatic increase in the number of filamented cells relative to the P_*marA*_-*cfp* strain (Fig. 2f).

### Filamented cells are able to regrow normally and are still susceptible to antibiotics

**Figure 3:**
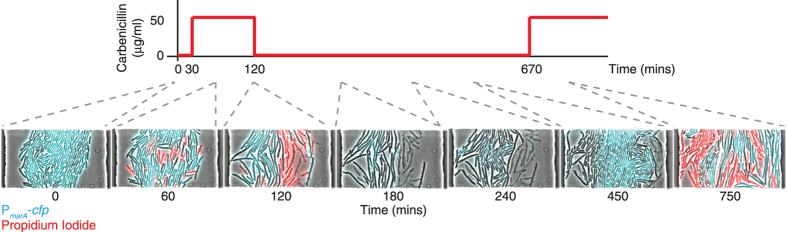
Resistance to carbenicillin is transient and cells that survive resume normal growth. Time-lapse microscopy images of P_*marA*_*-cfp* Δ*fliC* cells growing inside a microfluidic chamber subjected to two sequential steps of 50 μg/ml carbenicillin. Cyan indicates CFP levels from the MarA reporter; red indicates the death marker propidium iodide, which was added at the same time as carbenicillin. Supplementary Movie 3 shows additional details.

Are the filamented cells we observed following carbenicillin treatment able to resume growth after removal of carbenicillin? To test this, we used microfluidic chambers to trap cells while introducing and removing carbenicillin. Single cells were trapped in a microfluidic chamber and grown until the chambers were full. We then introduced a 90 min step of 50 μg/ml carbenicillin. Following this, we returned to conditions without the antibiotic, then later introduced a second step of carbenicillin (Fig. 3 and Supplementary Movie 3). As in our experiments on agarose pads with carbenicillin, we observed variability in MarA expression and heterogeneous responses, including both lysis and filamentation. Importantly, after carbenicillin was removed, the filamented cells were able to divide and regrow normally, suggesting the clinical relevance of transient antibiotic resistance. To confirm that these surviving cells were not resistant to antibiotics due to mutations or other non-transient mechanisms we introduced a second step of carbenicillin. We observed similar patterns of lysis and filamentation following this subsequent carbenicillin step, indicating that those cells that survived the first round of treatment were still susceptible to antibiotics.

### MarA levels stochastically exceed a threshold that confers transient resistance to carbenicillin

**Figure 4:**
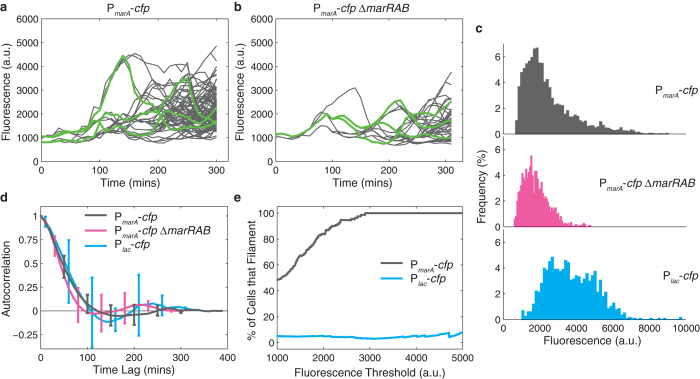
Level of MarA achieved by isogenic cells plays a key role in transient resistance to carbenicillin. (**a**) Representative fluorescence data extracted from a P_*marA*_*-cfp* microcolony. Gray traces show all cells within the microcolony, where branching indicates cell division. Green traces highlight representative lineages. (**b**) Representative fluorescence data for a P_*marA*_*-cfp* Δ*marRAB* microcolony. (**c**) Histograms showing frequency (%) of cells with a given fluorescence value. Data comes from six microcolonies for P_*marA*_*-cfp* and three microcolonies each for P_*marA*_*-cfp* Δ*marRAB* and P_*lac*_*-cfp*. (**d**) Autocorrelation of CFP signals for P_*marA*_*-cfp* (gray), P_*marA*_*-cfp* Δ*marRAB* (magenta), and P_*lac*_*-cfp* (cyan). For each, we calculated the average autocorrelation for all cells within a microcolony. Error bars represent the standard deviation across replicates, which are described above. (**e**) Percentage of filamented P_*marA*_*-cfp* (gray) and P_*lac*_*-cfp* (cyan) cells as a function of the fluorescence threshold level. The percentage is calculated as the number of filamented cells divided by the total number of filamented and lysed cells.

We were next interested in understanding how dynamic, heterogeneous MarA expression impacts survival. We first quantified the dynamics of P_*marA*_-*cfp* within microcolonies. We observed that MarA levels fluctuate in individual cell lineages (Fig. 4a). We also quantified fluorescence levels in the P_*marA*_-*cfp* Δ*marRAB* strain (Fig. 4b). Interestingly, the P_*marA*_-*cfp* Δ*marRAB* strain still exhibited fluctuations in MarA expression, though fluorescence levels were reduced relative to P_*marA*_-*cfp*. These residual dynamics could be due to the action of the MarA homologs Rob and SoxS^42^, other regulatory mechanisms that interact with P_*marA*_^43^, or dynamics intrinsic to the fluorescent reporter.

We conducted control experiments to eliminate the possibility that something about CFP expression or the *ssrA* tag was responsible for increasing antibiotic survival. To achieve this, we constructed a reporter strain that was independent of MarA where we could induce similar CFP expression levels to the P_*marA*_-*cfp* strain. We refer to this control strain as P_*lac*_-*cfp*. We observed variation in CFP expression across cell lineages, likely due to the intermediate induction levels (Supplementary Fig. 2a). We also measured the distribution of fluorescence levels for the P_*marA*_-*cfp*, P_*marA*_-*cfp* Δ*marRAB*, and P_*lac*_-*cfp* strains (Fig. 4c). CFP fluorescence for P_*marA*_-*cfp* had a long tail of high fluorescence values. For P_*marA*_-*cfp* Δ*marRAB* the distribution shape was similar, but the mean was slightly reduced and the tail of the distribution did not extend to CFP values that were as high as in the P_*marA*_-*cfp* strain. By design, the CFP levels for P_*lac*_-*cfp* were similar to or higher than those for P_*marA*_-*cfp*, but notably, the shapes of the distributions were different, suggestive of differing underlying dynamic processes^44^. To show that MarA levels, and not a reporter artifact, were causing heterogeneity in antibiotic survival, we placed P_*lac*_-*cfp* cells on agarose pads containing carbenicillin and recorded cell lysis and filamentation outcomes as before. In contrast to results with P_*marA*_-*cfp*, we did not observe a correlation between higher fluorescence levels and filamented cells (Supplementary Fig. 2b), confirming the contribution of MarA to heterogeneous antibiotic survival.

We measured the average autocorrelation of the CFP signal for P_*marA*_-*cfp*, P_*marA*_-*cfp* Δ*marRAB*, and P_*lac*_-*cfp* strains and observed no dominant periodicity in expression of any of the CFP signals (Fig. 4d). However, we note that although the average autocorrelation of CFP is similar between strains and is not indicative of a periodic signal, this does not preclude the possibility that stochastic properties differ, as these effects may be obscured by an average. Similar experiments with the MarA-CFP overexpression strain showed fewer fluctuations in fluorescence levels and slower dynamics, as expected due to the stabilized protein (Supplementary Fig. 3).

What is the relationship between cellular survival and fluorescence? Using the carbenicillin outcomes data (Fig. 2b and Supplementary Fig. 2b), we measured the percentage of cells above a threshold fluorescence level that filament upon carbenicillin treatment (Fig. 4e). As we increased the threshold for the P_*marA*_-*cfp* strain, a larger percentage of cells filamented, demonstrating a relationship between CFP levels and survival in these strains. The overall rate of filamentation in the P_*lac*_-*cfp* strain was lower than P_*marA*_-*cfp*, possibly due to differences in the reporter plasmid or induction conditions. However, in sharp contrast to P_*marA*_-*cfp*, the P_*lac*_-*cfp* strain always exhibited a constant percentage of filamented cells, regardless of the threshold fluorescence level we set. These results demonstrate that MarA expression is responsible for the increase in filamentation. Furthermore, the differences in cell lysis versus filamentation we observed between P_*marA*_-*cfp* and P_*marA*_-*cfp* Δ*marRAB* indicate that wildtype cells routinely exceed threshold levels of MarA required to provide transient resistance to lethal concentrations of carbenicillin, while Δ*marRAB* cells are far less likely to cross this threshold.

## Discussion

**Figure 5:**
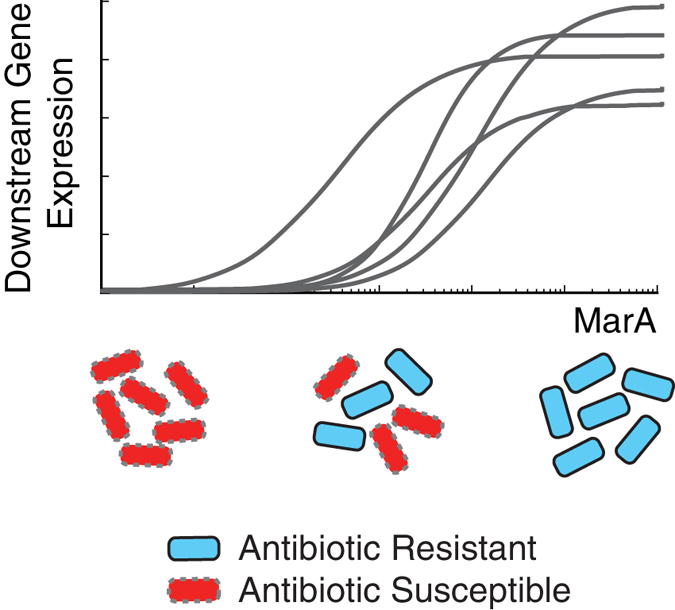
Transient resistance to antibiotics depends on MarA level achieved in the cell. Illustration showing expression of diverse downstream resistance genes as a function of MarA. Antibiotic susceptible cells are represented in red, resistant cells in cyan. As MarA levels increase, a larger number of downstream genes are turned on, providing antibiotic resistance. At low to intermediate levels of MarA, only a subset of the population has sufficient MarA, and consequently downstream gene expression, to ensure survival.

MarA activates a suite of downstream genes involved in antibiotic resistance^45^. Not all of these genes are activated at the same time or with the same number of MarA molecules^46^,^47^,^48^. Martin *et al.* estimated that, at a minimum, there is a 19-fold difference in the number of MarA proteins needed for half-saturation of the different downstream promoters^46^. Furthermore, physiological levels of MarA are far lower than those required to achieve saturation for the majority of these downstream genes^46^. Our findings suggest a possible mechanism for how cells within a population could achieve a gradient of resistance levels in a transient fashion. Cells that stochastically express higher levels of MarA may transiently turn on more downstream genes than those with low levels of MarA, leading to elevated resistance (Fig. 5). Because MarA expression is dynamic, a cell with high MarA that expresses many downstream genes will eventually revert to conditions with lower levels of resistance. Stochastic expression of a transcriptional factor could serve to coordinate expression of multiple downstream genes simultaneously^49^. When we conducted experiments in the Δ*marRAB* strain, cells still exhibited dynamic expression of P_*marA*_-*cfp*, however carbenicillin survival rates were reduced, suggesting that variability in MarA expression in wildtype cells is sufficient to allow a subset of the population to transiently achieve levels required for antibiotic resistance.

The genetic mechanism that underlies stochastic expression of MarA is an interesting area for future study. A computational model from our group proposed a mechanism involving feedback control of *marRAB* expression that can lead to stochastic dynamics^37^. However, our results here suggest that this model is incomplete, as cells retain dynamic behavior even when the *marRAB* operon is deleted. It is possible that other regulatory proteins, such as SoxS and Rob play a complementary role, or that degradation of these proteins by Lon protease may introduce additional dynamics^41^,^42^. Indeed, MarA does not function alone in the cell but together with SoxS and Rob. These three regulators control a common set of downstream genes and significant interactions have been identified between them^42^. Although we have focused here on the uninduced case, a recent stochastic modeling study suggests that induction of MarA expression may have interesting dynamics^50^. Time-lapse experiments could explore the effect of induction on the network at the single cell level. In addition, it will be interesting to explore the genetic basis for the long tail of MarA levels observed in the P_*marA*_-*cfp* strain (Fig. 4c), which may be the result of the feedback architecture controlling expression of the *marRAB* operon.

Expressing downstream genes involved in antibiotic resistance imposes a burden on cells^51^. Generating diverse resistance phenotypes within a population could serve as a bet hedging strategy to allow populations to survive antibiotic treatment without requiring that all cells express these costly genes. Similar strategies have been demonstrated at the single cell level through studies on bacterial persistence^3^,^6^,^24^. Our findings present an alternative to dormancy, where cells temporarily grow into filaments and continue producing cellular components^39^. Other studies have pointed to alternative mechanisms by which cells can generate a continuum of transient resistance levels. For example, resistance has been shown to be negatively correlated with RpoH expression in *E. coli*^19^, positively correlated with the cephalosporin hydrolase gene in *E. coli*^20^, and with the porin gene *ompC* in *S. typhimurium*^21^. These studies, coupled with our findings, suggest that it is important to consider stochastic, single cell level effects associated with expression of antibiotic resistance genes and their regulators.

Stochastic gene expression can facilitate evolutionary adaptation^52^. A recent study showed that antibiotic resistance can emerge from multinucleated bacterial filaments via the SOS response^53^. A strategy where a subset of cells have high MarA levels and grow into filaments could generate a favorable environment for mutations, especially over long time or cyclic exposure to antibiotics. We have identified a new stochastic role for the multiple resistance activator MarA, where even without induction, a subset of cells achieve expression levels sufficient to achieve transient resistance to antibiotics.

## Methods

### Plasmids and Strains

To construct P_*marA*_-*cfp* we placed a modified *marRAB* promoter upstream of a degradation tagged *cfp*. We used a copy of the wildtype *marRAB* promoter and introduced transversion mutations to inactivate the two MarR binding sites^29^. This plasmid was transformed into *E. coli* MG1655.

The P_*marA*_-*cfp* Δ*marRAB* strain is *E. coli* MG1655 Δ*marRAB* transformed with the plasmid described above.

The MarA-CFP translational fusion strain is *E. coli* MG1655 transformed with a plasmid where *marA* is fused to an untagged version of *cfp* using a (Gly_2_-Ser)_2_ linker downstream the lacUV5 promoter.

The P_*lac*_-*cfp* strain is *E. coli* MG1655 transformed with a plasmid where degradation tagged *cfp* is fused to the lacUV5 promoter.

Further details on plasmid and strain construction are provided in Supplementary Information.

### Time-lapse Microscopy

Overnight cultures were grown from single colonies in LB medium with 30 μg/ml kanamycin. From these cultures a 1:100 dilution was used to inoculate fresh LB containing 30 μg/ml kanamycin (for MarA-CFP and P_*lac*_-*cfp* experiments, this was supplemented with 100 μM or 50 μM IPTG, respectively). Cultures were incubated for 3 hrs at 37 °C with shaking. Cells were then diluted 1:100 in M9 minimal medium containing 0.2% glycerol, 0.01% casamino acids, 0.15 μg/ml biotin, and 1.5 μM thiamine (which we denote MGC medium). Cells were then placed on 1.5% MGC low melting temperature agarose pads containing kanamycin and IPTG as described above. Cells were imaged at 100× using a Nikon Instruments Ti-E microscope. The temperature of the microscope chamber was held at 32 °C for the duration of the movies.

For the single cell carbenicillin assays, cells were prepared as described above, but were diluted 1:3 in MGC following the 3 hr incubation. 50 μg/ml carbenicillin and 10 μg/ml propidium iodide were added to the agarose pads.

For the microfluidic chip experiments, we used P_*marA*_-*cfp E. coli* MG1655 Δ*fliC*; the *fliC* deletion makes the strain non-motile. 50 μg/ml carbenicillin and 10 μg/ml propidium iodide were introduced at the times shown in Fig. 3.

Image analysis was performed using custom MATLAB software.

See Supplementary Information for full methods.

## Additional Information

**How to cite this article**: El Meouche, I. *et al.* Stochastic expression of a multiple antibiotic resistance activator confers transient resistance in single cells. *Sci. Rep.* **6**, 19538; doi: 10.1038/srep19538 (2016).

## Supplementary Material

Supplementary Information

Supplementary Movie 1

Supplementary Movie 2

Supplementary Movie 3
