# Supplementary material for: Stochastic expression of a multiple antibiotic resistance activator confers transient resistance in single cells
Source: Sci Rep. 2016 Jan 13;6:19538. doi: 10.1038/srep19538 (PMC4725842; doi:10.1038/srep19538)
Supplement: Supplementary Information [file srep19538-s1.pdf]

SUPPLEMENTARY INFORMATION

Stochastic expression of a multiple antibiotic resistance activator confers transient resistance in single cells

Imane El Meouche, Yik Siu, Mary J. Dunlop

SUPPLEMENTARY METHODS

Plasmids and Strains

The strains used in this study were all derived from *E. coli* K-12 MG1655 [1].

P_{marA}-cfp

To construct the MarA reporter plasmid, *cfp* was derived from [2] and was tagged with an *ssrA* tag consisting of TSAANDENYALAA [3]. The TS sequence acts as a linker and the *ssrA* tag (underlined) increases the degradation rate of CFP, providing increased dynamic resolution.

We used a copy of the wildtype *marRAB* promoter and introduced transversion mutations to inactivate the two MarR binding sites. The transversion mutations were derived from [4], where they are listed as “TV -14 to -18” and “TV +11 to 15”. The MarA binding site is shown in italics, the former MarR binding sites are underlined, and the transversion mutations are shown in bold.

TGCATCGCATTGAACAAAACTTGAACCGAT*TTAGCAAAACGTGGCA*TCGGTCA
ATTCATTCATTTGACTTATACTTGCCTG**TTACC**TATTATCCCCTGCAACTAATT
AC**GGTAA**AGGGCAACTAATGTGAAAAGTACCAGCGATCTG

This sequence was cloned in place of the promoter in the low-copy (SC101) origin vector pBbS5k [5] to control expression of the tagged *cfp*. We also removed the extraneous copy of *lacI* and its promoter from the pBbS5k plasmid. This plasmid was transformed into *E. coli* MG1655.

P_{marA}-cfp *Δ*marRAB

This strain uses the MarA reporter plasmid described above, transformed into *E. coli* MG1655 Δ*marRAB*, which is described below.

MarA-CFP Translational Fusion

To construct the protein fusion of MarA and CFP, we amplified the *cfp* gene from [2] using the forward primer ATGACTAGCAAAAGAAGC and the reverse primer. CTATTATTTATACAGTTCATCCATG. We inserted a (Gly$_2$-Ser)$_2$ linker sequence between the *marA* and *cfp* genes [6]. *marA* was amplified from *E. coli* MG1655 genomic DNA, starting at the start codon and stopping immediately before the *marA* stop codon. The translational fusion was cloned into the medium copy (p15A) origin vector pBbA5k [5]. This plasmid was transformed into *E. coli* MG1655.

P_{lac}-cfp *MG1655*

cfp tagged with an *ssrA* tag (as described above) was cloned in the low-copy (SC101) origin vector pBbS5k [5]. This plasmid was transformed into *E. coli* MG1655.

Deletion of marRAB *Operon and* fliC *Gene*

In order to delete the *marRAB* operon and *fliC* gene, we used homologous recombination [7]. We generated PCR products using primers with extensions homologous to the regions adjacent to the operon or gene.

For the *marRAB* operon, we used the forward primer:
CCAGCGATCTGTTCAATGAAATTATTCCATTGGGTCGCTTAATCCATATGGTGTAGGCTGGAGCTGCTTC
and the reverse primer:
AAGCCCCGAGATGTCGGGGCCAGAACAAACTACATAGCGTGTTGATTATAATTCCGGGGATCCGTCGACC.

For the *fliC* gene, we used the forward primer:
CCCTGCAGCAGAGACAGAACCTGCTGCGGTACCTGGTTAGCTTTTGCCAAGTGTAGGCTGGAGCTGCTTC
and the reverse primer:
ATGGCACAAGTCATTAATACCAACAGCCTCTCGCTGATCACTCAAAATAAATTCCGGGGATCCGTCGACC.

These primers were used to amplify a kanamycin resistant gene flanked by FRT regions from pKD13 [7]. Bold letters indicate the homologous recombination extensions. We followed the protocol for recombination in [7] using the phage lambda recombinase harbored on the low copy plasmid pSIM6. After recombination, the resistance marker gene was removed using the pCP20 helper plasmid encoding the FLP recombinase. To verify the insertion and removal of the resistance gene, we used a reverse primer from the resistance gene, K2: CGGTGCCCTGAATGAACTGC and a forward primer in the region upstream of the *marRAB* operon: ATAAGGGGTAAACAAGGATAAA or the *fliC* gene: CAGGCAATTTGGCGTTGCCG. Reverse primers downstream of the *marRAB* operon: ACTATTGTTGGATGAACGCT or the *fliC* gene: CCAATACGTAATCAACGACTTGC were used to verify deletions in the mutant strains.

Determining the Minimum Inhibitory Concentration [8] of Carbenicillin

Overnight cultures of P_{marA}-*cfp* and MarA-CFP were diluted 1:100 in LB medium supplemented with 30 μg/ml kanamycin (and 100 μM IPTG for MarA-CFP). Then, the diluted cultures were incubated at 37°C for 8 hours with shaking. The OD_{600} of each culture was normalized to start new cultures in the presence of antibiotics. Cells were incubated with increasing concentrations (0 to 100 μg/ml) of carbenicillin at 37°C for 20 hours with shaking. The final OD_{600} was then determined and cell growth was considered inhibited if the final OD_{600} measured was lower then 0.1.

Determining the Half-life of MarA-CFP

MarA-CFP overnight cultures were diluted 1:100 in 30 ml LB medium supplemented with 30 μg/ml kanamycin and 100 μM IPTG. Expression was induced for 4 hours and then chloramphenicol (100 μg/ml) was added to the cultures to stop protein synthesis. 1 ml of the culture was collected and transferred at different time points (0, 5, 10, 15, 30, and 60 mins). In order to extract total proteins, cells were harvested at 13,000 rpm for 3 mins and

the supernatant was removed. The cell pellets were resuspended with 4% SDS and shaken for 15 mins at 37°C. Glass beads (0.1 mm) were added and samples were vortexed for 10 sec and incubated on ice for 10 sec three times. The samples were then centrifuged and the supernatant containing the total proteins was collected. Proteins were then normalized using the Pierce 660nm Protein Assay Reagent (Fisher) according to the manufacturer's recommendations. After separation by SDS-PAGE, proteins were electroblotted (Bio-Rad) onto Hybond-enhanced chemiluminescence (ECL) nitrocellulose membranes (Fisher). Membranes were probed first with 1:3000 rabbit anti-MarA antibodies generously provided by Valerie Duval, Laura McMurry, and Stuart Levy [9]. Primary antibodies were detected using the secondary Peroxidase-AffiniPure Goat Anti-Rabbit IgG (Jackson ImmunoResearch). Immunodetection of proteins was performed with the SuperSignal West Femto Kit (Fisher) according to the manufacturer's recommendations. Blots were scanned using the VersaDoc 4000 MP (Bio-Rad).

Microfluidic Experiment

For these experiments we used the strain *E. coli* MG1655 Δ*fliC* transformed with the plasmid described in P_{marA}-*cfp* above. The *fliC* deletion renders the strain non-motile, which we found to be essential for conducting experiments using microfluidic chips (motile cells swim out of the trapping chambers). The *fliC* deletion had no impact on the minimum inhibitory concentration of carbenicillin in our conditions.

An overnight culture of P_{marA}-*cfp* Δ*fliC* was diluted 1:1000 in 50 ml LB medium with 30 μg/ml kanamycin. When the OD_{600} reached 0.2-0.3, cells were harvested by centrifugation at 2700 x g for 10 mins and resuspended in 1 ml LB medium with 0.075% Tween 80 (Sigma-Aldrich) and 30 μg/ml kanamycin. The resuspended cells were introduced into the microfluidic device through the cell port, as described in [10]. Once cells were trapped inside the chamber, fresh LB medium with 0.075% Tween 80 and 30 μg/ml kanamycin was flushed through the media channels outside the cell-trapping chambers to supply cells with continuous nutrients. After cells grew to fill the chambers (approximately 4 hours), we began imaging. We denote this time t = 0 mins. The first carbenicillin step was introduced at t = 30 mins, then removed at t = 120 mins. The second step was introduced at t = 670 mins and lasted until the end of the experiment. The addition and removal of carbenicillin was achieved by switching the positions of the syringe media reservoirs on the syringe tower (Quantitative BioSciences, Inc. and described in [10]), in which one of the syringes contained LB medium with 0.075% Tween 80 and 30 μg/ml kanamycin while the other contained LB medium with 0.075% Tween 80, 30 μg/ml kanamycin, 50 μg/ml carbenicillin, and 10 μg/ml propidium iodide. Phase contrast, CFP, and RFP images were taken every 3 minutes for the duration of the experiment.

Microfluidic Device Fabrication

We obtained the silicon wafer used for microfluidic device fabrication from Quantitative BioSciences, Inc. with feature design published previously [10,11]. The device was built using standard soft lithography methods [10]. First, polydimethylsiloxane (PDMS) was made by mixing the base and curing agent of the Ellsworth Sygard 184 Silicone Kit (Fisher) in a ratio of 10:1. Then, the PDMS was degassed inside a desiccator connected to a vacuum pump. The degassed PDMS was then poured onto the silicon wafer followed by an extra

degassing step to remove the air bubbles formed during pouring. Next, the PDMS was incubated at 75°C for 2 hours and was subsequently removed from the wafer. Individual PDMS devices were cut out using a razor blade. Each port of the device was made accessible to the connection pin by punching through the PDMS using a 22-gauge leur stub (McMaster-Carr). Then, the ports of the PDMS device were washed with DI water to remove any PDMS debris inside, and the exterior of the PDMS device was cleaned with 70% ethanol and rinsed with deionized water. The cleaned PDMS devices were incubated at 75°C overnight to remove any residual water. The dried devices were cleaned with scotch tape to remove any debris on the surface of the PDMS. Then, the PDMS device and a clean glass cover slip were treated using a BD-20AC Laboratory Corona Treater (Electro-Technic Products) for ~30 seconds and bonded with each other. After bonding, the microfluidic devices were incubated at 75°C overnight to facilitate the formation of the covalent bond between the PDMS and the glass cover slip.

Autocorrelation Calculations

The autocorrelation of the CFP signal, f(t), is given by

$$R(\tau) = \frac{1}{N-\tau}\sum_{n=0}^{N-\tau-1} \tilde{f}(n+\tau)\tilde{f}(n)$$

where

$$\tilde{f} = f - \frac{1}{N}\sum_{n=1}^{N-1} f(n)$$

τ is the lag time, and N is the number of time points. This function was then normalized so that the value of $R(0) = 1$.

We used a modified version of this standard formula to avoid overcounting of branched data. When a mother cell divides into two daughter cells, the overcounting correction removes one instance of the contribution from the mother's portion of the lineage, which is common between the daughters. Thus, the initial contribution is only counted once and not twice. Details of the modified autocorrelation formula are available in [2].

SUPPLEMENTARY FIGURES

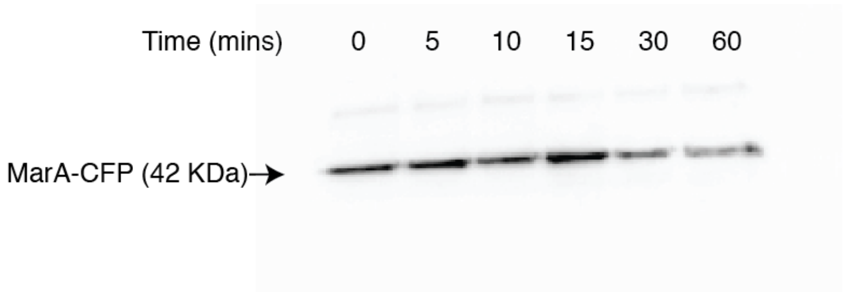

Supplementary Figure 1: The fused protein MarA-CFP has a ~30 min half-life. To determine the half-life of MarA-CFP, we overexpressed it from an IPTG inducible promoter and added chloramphenicol to arrest protein synthesis. Western blot analysis shows MarA-CFP levels at different times, detected using an anti-MarA antibody. Total protein level was normalized between samples. These results are a representative example of one of three biological replicates.

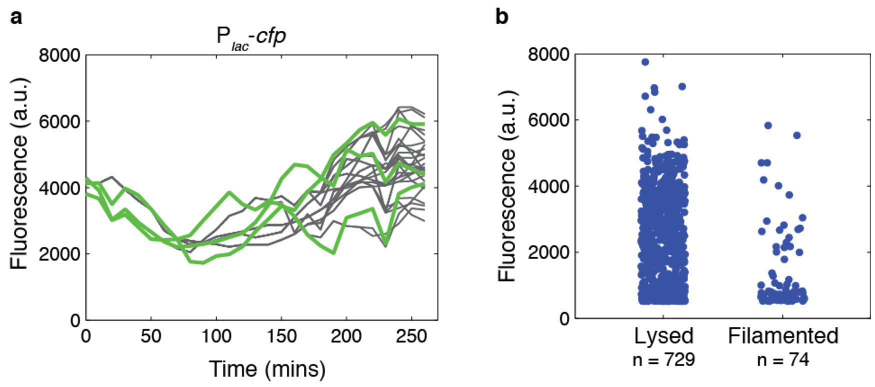

Supplementary Figure 2: Inducible fluorescent reporter does not show a correlation between fluorescence levels and carbenicillin survival. (a) Extracted fluorescence data for a microcolony expressing the P_{lac}-*cfp* reporter. Each gray line corresponds to a single cell lineage and green lines highlight representative lineages. (b) Outcomes of P_{lac}-*cfp* cells after 400 mins of exposure to 50 µg/ml carbenicillin. Each blue dot corresponds to one cell, which has an outcome of 'lysed' or 'filamented' and an initial fluorescence value. The number of cells exhibiting each outcome is listed on the x-axis.

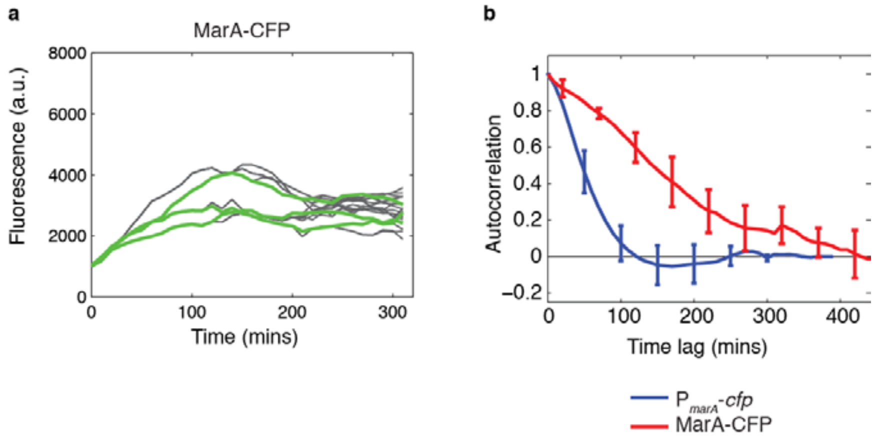

Supplementary Figure 3: MarA-CFP protein fusion has low cell-to-cell variability. (a) Extracted fluorescence data from a microcolony expressing the MarA-CFP reporter. Each gray line corresponds to a single cell lineage and green lines highlight representative lineages. (b) The average autocorrelation of fluorescence levels shows differences in dynamics between P_{marA}-*cfp* (blue) and MarA-CFP (red). Error bars show standard deviation across six microcolonies for P_{marA}-*cfp* and three microcolonies for MarA-CFP. Note that the longer autocorrelation times for MarA-CFP are expected due to slower growth of this strain and the stabilized CFP reporter.

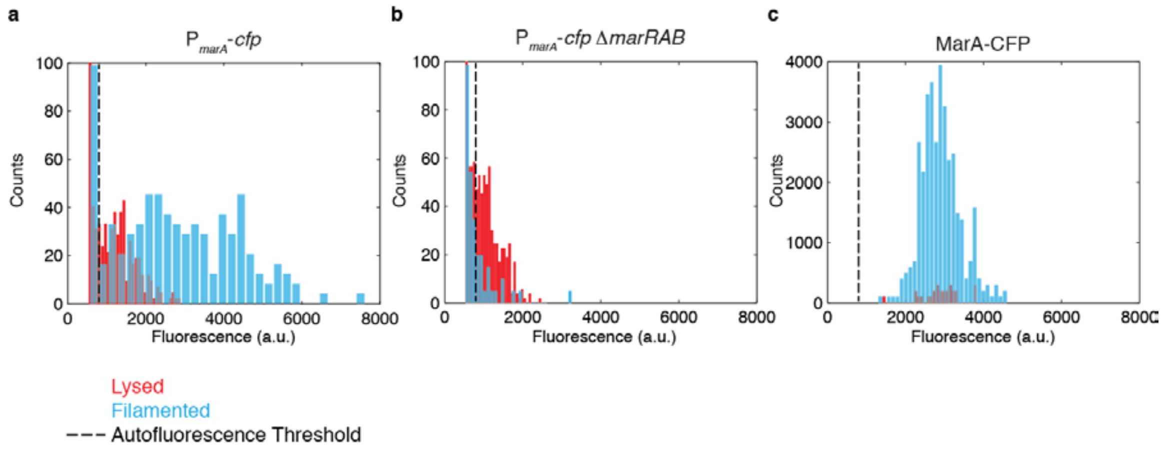

Supplementary Figure 4: P$_{marA}$-*cfp* lysed and filamented cell distributions have statistically different means. Data from Fig. 2b, d, and f plotted as histograms with lysed cell distributions in red and filamented in cyan: (a) P$_{marA}$-*cfp*, (b) P$_{marA}$-*cfp* Δ*marRAB*, and (c) MarA-CFP. In our microscopy images some cells were at or near the autofluorescence level, creating an artificial peak in the data at low fluorescence values for some of the strains. To account for this, we set a threshold, shown with a black dashed line (fluorescence = 800 a.u.), and included only data that exceeded this threshold in our statistical tests. Only the P$_{marA}$-*cfp* lysed and filamented cell distributions have statistically different means ($P < 0.01$ by a Mann-Whitney rank sum test). We note that results are not sensitive to the exact threshold value used.

SUPPLEMENTARY MOVIE LEGENDS

Supplementary Movie 1: Time-lapse movie showing cell-to-cell variability of MarA expression. Representative time-lapse microscopy movie of P_{marA}-*cfp*. Images were taken every 10 mins. CFP levels are indicated in cyan.

Supplementary Movie 2: Time-lapse movie showing heterogeneous survival in response to carbenicillin. Representative time-lapse microscopy movie of P_{marA}-*cfp* with 50 μg/ml carbenicillin exposure. Images were taken every 10 mins. CFP levels are indicated in cyan; propidium iodide levels are indicated in red.

Supplementary Movie 3: Time-lapse microscopy showing repeated carbenicillin treatment of cells growing in microfluidics chambers. Representative time-lapse microscopy movie of P_{marA}-*cfp* Δ*fliC* growing in a microfluidic chamber with repeated exposure to carbenicillin. Cells were grown in a constant flow of LB medium with two exposures to 50 μg/ml carbenicillin, one from t = 30 - 120 mins and one from t = 670 mins until the end of the movie. 10 μg/ml propidium iodide is added at the same time as carbenicillin. CFP levels are indicated in cyan; propidium iodide levels are indicated in red.

REFERENCES

1 Blattner, F. R. *et al.* The complete genome sequence of *Escherichia coli* K-12. *Science* **277**, 1453-1462 (1997).

2 Dunlop, M. J., Cox, R. S., Levine, J. H., Murray, R. M. & Elowitz, M. B. Regulatory activity revealed by dynamic correlations in gene expression noise. *Nature Genetics* **40**, 1493-1498 (2008).

3 Andersen, J. B. *et al.* New unstable variants of green fluorescent protein for studies of transient gene expression in bacteria. *Applied and environmental microbiology* **64**, 2240-2246 (1998).

4 Martin, R. G. & Rosner, J. L. Transcriptional and translational regulation of the *marRAB* multiple antibiotic resistance operon in *Escherichia coli*. *Molecular microbiology* **53**, 183-191, doi:10.1111/j.1365-2958.2004.04080.x (2004).

5 Lee, T. S. *et al.* BglBrick vectors and datasheets; a synthetic biology platform for gene expression. *Journal of Biological Engineering* **5**, 12 (2011).

6 Klein, J. S., Jiang, S., Galimidi, R. P., Keeffe, J. R. & Bjorkman, P. J. Design and characterization of structured protein linkers with differing flexibilities. *Protein engineering, design & selection : PEDS* **27**, 325-330, doi:10.1093/protein/gzu043 (2014).

7 Datsenko, K. A. & Wanner, B. L. One-step inactivation of chromosomal genes in *Escherichia coli* K-12 using PCR products. *Proceedings of the National Academy of Sciences of the United States of America* **97**, 6640-6645, doi:10.1073/pnas.120163297 (2000).

8 Wall, M. E., Markowitz, D. A., Rosner, J. L. & Martin, R. G. Model of Transcriptional Activation by MarA in *Escherichia coli*. *PLoS computational biology* **5**, e1000614 (2009).

9 McDermott, P. F., White, D. G., Podglajen, I., Alekshun, M. N. & Levy, S. B. Multidrug resistance following expression of the *Escherichia coli marA* gene in *Mycobacterium smegmatis*. *Journal of bacteriology* **180**, 2995-2998 (1998).

10 Ferry, M. S., Razinkov, I. A. & Hasty, J. Microfluidics for synthetic biology: from design to execution. *Methods Enzymol* **497**, 295-372, doi:10.1016/B978-0-12-385075-1.00014-7 (2011).

11 Mondragon-Palomino, O., Danino, T., Selimkhanov, J., Tsimring, L. & Hasty, J. Entrainment of a population of synthetic genetic oscillators. *Science* **333**, 1315-1319, doi:10.1126/science.1205369 (2011).
